# Women’s perspectives of decision-making for labour and birth: a qualitative antenatal-postnatal paired interview study

**DOI:** 10.1136/bmjopen-2024-096171

**Published:** 2025-06-04

**Authors:** Erin White, Anna Davies, Andrew Demetri, Sheelagh McGuinness, Gemma Clayton, Abigail Fraser, Sonia Barnfield, Danya Bakhbakhi, Emma Claire Anderson, Katherine Birchenall, Rachel Miller, Christy Burden, Abi Merriel, Carol Kingdon

**Affiliations:** 1 Department of Women’s & Children’s Health, University of Liverpool, Liverpool, UK; 2 University of Bristol, Bristol, UK; 3 Department of Women's and Children’s Health, North Bristol NHS Trust, Bristol, UK; 4 N/A, Patient Representative, Bristol, UK

**Keywords:** Pregnancy, Decision Making, Natural Childbirth

## Abstract

**Abstract:**

**Objectives:**

To understand and compare women’s antenatal and postnatal views on: (1) priorities for information provided about labour and delivery and (2) decision-making in labour and delivery.

**Design:**

Qualitative interview study using repeat interviews at two time points: during pregnancy (≥13 weeks gestation); and after birth (≥6 weeks).

**Setting:**

Large maternity hospital in the Southwest of England.

**Participants:**

Pregnant women accessing antenatal care were purposively sampled and recruited antenatally by community midwives to ensure representation from different sociodemographic groups, with diverse experiences of low and high-risk care.

**Data collection:**

Telephone interviews with a single researcher using a semistructured interview topic guide.

**Data analysis:**

Interviews were audio recorded, transcribed verbatim, and qualitative thematic analysis was conducted using Braun and Clarke’s six-stage process.

**Results:**

Twelve women participated (12 antenatal interviews; 10 follow-up postnatal interviews). Overall, women’s postnatal views were consistent with their antenatal views about what they wanted to know and the factors that influence decision-making. Three themes were generated. Theme 1 ‘Sources of information’ presents evidence of how women obtain and use information (sub-themes: ‘social influences’, ‘patient responsibility for information seeking’, ‘NHS vs non-NHS resources’). Theme 2 reports women’s views and experiences of ‘The influence of Healthcare Professionals in decision-making’ (sub-themes ‘patient and professional roles in decision-making’, ‘conflicting advice and preferences’, ‘taking authority in emergency decision-making’). The final theme, theme 3, ‘When, how, and what information women want’ shows women want time to process information (sub-themes ‘when: it’s definitely information and time’, ‘how: presentation of information’, ‘what: information required’). Cross-cutting all themes, we found an unmet need for information to be tailored to the individual.

**Conclusions:**

Women understand decision-making during labour and birth is a dynamic process. Women can struggle with the volume, quality and timing of information available. In busy maternity settings, the challenge is to better equip women with the information they want, and health professionals with the information they need to provide for personalised care and shared decision-making. Antenatal interventions that warrant further research include decision aids, birth plans, and structured counselling using core information sets. Insights from both antenatal and postnatal perspectives will help inform their development.

**Strengths and Limitations of this study**

- Paired interview design capturing rich data on women’s views of information quality, decision-making encounters, and experiences of actual labour and birth.
- Patient and public representatives contributed to protocol development and participant materials including information about the study.
- Recruitment was limited to a single healthcare Trust; however, diverse participant experiences were obtained from in-depth interviews.
- This article focuses on women’s perspectives of shared decision-making; professional perspectives are reported in a companion study. 10.1136/bmjopen-2023–0 80 961

## Introduction

 Informed decision-making (IDM) is one of the foundations of patient autonomy and consent within medicine.^1 2^ Shared decision-making (SDM) involves a discussion between patients and professionals about available options, taking into account the best available evidence and the patient’s context, values and preferences to enable them to make an informed choice about care.^3^

In maternity care, decision making can be challenging. Labour and birth are dynamic and often demand decisions to be made during the process, rather than at the outset. This decision-making is further complicated because two individuals are involved (the woman and foetus). Further layers of complexity are added if a woman’s pre-specified choices are no longer an option and clinicians must offer alternative care. Decision-making about mode of birth,^4^,^6^ pain relief^7^,^10^ and assisted delivery^11 12^ can arise during labour. When intervention is needed, some women wish to retain a sense of control over decision-making.^13^ This can be especially difficult in time-critical emergencies.^14 15^ Yet not informing or involving women in decisions made can impact women physically and psychologically, contributing to trauma or amount to negligence.^16 17^ SDM can enable safer care and improve birth experiences for women.^18^

In the United Kingdom (UK), the 2015 landmark *Montgomery vs Lanarkshire* legal case has been a catalyst for efforts to improve SDM.^19 20^ The *Montgomery* test requires healthcare professionals (HCPs) to inform patients of any material risks and appropriate alternatives prior to treatment, enabling patients to make well-informed decisions.^19^ However, maternity HCPs report limited training and require additional support to deliver better quality information in the current care context.^21^ Meanwhile, women describe wanting more information and express a desire to have been told it before birth to help them manage expectations and make decisions in all potential labour and birth scenarios.^22^,^24^ Antenatal information can assist in managing expectations and address uncertainties to improve SDM during labour and birth.^22 25^ However, discrepancies may exist in what women think they want to know before birth and what they wish they had known after their birth.^16 26^ For example, antenatally women often underestimate the need for pain relief, and postnatally express their need for additional analgesia.^26^ Addressing this expectations-experience gap through the provision of information is further complicated if women express antenatally that they would rather not know or are not open to other options.^16^ Postnatally, this can cause dissatisfaction and increased risk of post-traumatic stress disorder (PTSD).^16 27^

To improve SDM, IDM and overall birth experiences, it is therefore important to understand what antenatal information women require, and how this could best be presented to support ongoing individual decision-making during labour and birth. There is a need to address the gap in current knowledge about how antenatal information requirements translate to postnatal reflections on actual information needs if we are to improve the quality of information provided for SDM. This study aims to identify and compare antenatal and postnatal perceptions of information provision and decision-making for labour and birth by analysing paired interview data from the same women conducted before and after delivery.

### Terminology

This study was developed at a time of controversy surrounding the language used in maternity care.^28 29^ We use the term ‘shared decision-making’ after asking participants about their understanding of the term as previously defined.^30 31^ We understand terms such as ‘informed consent’, ‘informed decision-making’ or ‘supported decision-making’ are now used as alternatives.^29 32 33^ We therefore also use ‘decision-making’ as an umbrella term to include all experiences and forms of decision-making. We also use the terms ‘woman/women’. This study involved people who identified as women; therefore, this terminology accurately references the study findings. We do, however, acknowledge the use of other inclusive terminology that incorporates the pregnant person.

## Methods

This was an interpretivist qualitative study using semi-structured interviews.^34^ The Standards for Reporting of Qualitative Research (SRQR) checklist was used^35^ (online supplemental table S1). This study is part of a suite of studies aimed at improving how information is provided to women about interventions during birth to improve their preparation for intrapartum interventions, their experience of the consent process, and ultimately improve their birth experience.^21 36^

### Patient and public involvement

Two patient representatives contributed to protocol development and participant information materials and topic guides.

### Research team and reflexivity

The research team includes practising obstetricians (AM, CB), trainee obstetrician (ADe), health psychologists (ADa, EA), statistician (GC), epidemiologist (AF), legal academic (SMcG), medical student (EW) and a sociologist (CK). Interviews were conducted by ADa. Analysis of interview transcripts was carried out by EW, AM and CK. Discussion, reflection and subsequent agreement of identified themes and subthemes by the research team, from their varied backgrounds, aimed to neutralise personal biases about SDM.^37 38^ We acknowledge personal and interpersonal reflexivity and how our clinical or academic backgrounds may have introduced bias in data collection and interpretation.^37^ To mitigate this, the multiple perspectives employed during the thematic analysis aimed to challenge assumptions and decisions,^37^ ensuring a more balanced and comprehensive understanding of the data.

### Study design

We explored women’s views and experiences antenatally and postnatally in semi-structured interviews. We interviewed the same women, at two time points, before and after birth, to ascertain what women think they need to know, what they wish they had known, and what influenced actual decision-making during labour and birth. As a method, repeat interviews are not new,^39^ especially in qualitative and mixed-method longitudinal studies of birth.^39^,^41^ Postnatal reflections can provide insight into information requirements for labour and birth,^23^ but alone can be subject to recall bias and may not represent women’s antenatal views.^16^ Repeat interviews can elicit more nuances, ambiguity and complexity than a single interview.^42^

### Participant selection, sampling and sample size

Participants were purposively sampled from a single NHS Trust in the Southwest of England, with approximately 6000 deliveries per annum. Women had to be pregnant (>12 weeks gestation), planning to receive intrapartum care from the Trust (in a hospital or community setting), aged 18 years or over, and English speaking to be eligible for inclusion. Exclusion criteria included women unable to give informed consent (online supplemental table S2). We sought breadth and depth of views with representation from diverse women according to socio-economic background, parity, previous birth experience and complexity of current pregnancy. We approached both low and high-risk antenatal women in their first or subsequent pregnancy. Potential participants received study information from community midwives, in antenatal clinics and on antenatal wards, which included a contact for the study team who then screened and arranged an individual interview from 12 weeks’ gestation. A second postnatal interview was arranged around 6 weeks postnatally. Participants received a £10 voucher for participation. Recruitment continued until the research team agreed that no new themes were arising in the phase 1 interviews, suggesting that appropriate data saturation for answering the main question was reached.^43^

### Data collection

The semi-structured topic guide explored decision-making for labour and birth, consent, knowledge of birthing interventions and options (online supplemental file S3: Semi-structured Interview Guide). All interviews were conducted by telephone and written informed consent was gained prior. Interviews were audio recorded using an encrypted device, transcribed verbatim and pseudonymised.

### Data analysis

Braun and Clarke’s six-stage method of thematic analysis was used (online supplemental file S4).^44^,^46^ The process initially involved reading the transcripts several times to identify important patterns in the data and generate initial codes.^46 47^ Transcripts were read both in chronological groups (timepoint 1, timepoint 2) and individually (antenatal, postnatal) for comparisons (EW, AM, CK). Transcripts were read first by timepoint to identify common themes. All antenatal and postnatal interview data was coded individually using Microsoft Excel (EW).^46 47^ Codes were then examined across the chronological groups before the consolidation of themes. Initial codes were then grouped to generate six initial themes (EW).^46 47^ Themes were revisited to check if there was enough evidence to support them, to check if themes were too similar and should be collapsed, and to check for disconfirming evidence (EW, AM, CK). Themes were revised accordingly with three final themes generated and nine sub-themes (EW, AM, CK).^46 47^ For trustworthiness, the familiarisation of data and final generation of themes was carried out independently by three authors and then reviewed collaboratively (EW, AM, CK).^46 47^ All authors independently agreed that sufficient data was achieved to claim saturation.^48^

## Results

Twelve women completed antenatal interviews (between October 2021 and March 2022), ten of whom also gave postnatal interviews (between January 2022 and May 2022). Interviews lasted approximately sixty minutes. A total of 46 women expressed an interest in participating, 20 of whom were contactable (after three attempts) and received a participant information leaflet. Eight of these women were not interviewed for reasons that included developing pregnancy complications and/or giving birth, making them ineligible. Table 1 summarises participant characteristics, preferences, and experiences. Participants were predominantly primiparous women (with only one multiparous woman), likely because they were less well-informed, had not previously experienced childbirth, and may have had more availability to participate in interviews. Participants also represented diverse pregnancy and birth experiences, including both singleton and multiple pregnancies.

**Table 1:** Summary of paired antenatal and postnatal interview participants

Participant pseudonym	Antenatal interview				Postnatal interview		
	Trimester	Parity	Pregnancy	Mode of birth preference	Obstetric factors	Actual birth mode	Gestation
Amie (002)	Third trimester	Primip	Singleton	Ambivalent/ELCS	Induction of labour	EMCS	Term
Hannah (004)	Third trimester	Primip	Singleton	Vaginal	Spontaneous rupture of membranes	Vaginal	Term
Joanne (005)	Second trimester	Primip	Multiples (twins)	ELCS	Preterm labour (twins)	EMCS Cat 1	Pre-term
Vicky (007)	Third trimester	Primip	Singleton	Vaginal/ELCS for breech	Breech presentation	ELCS	Term
Stephanie (008)	Third trimester	Primip	Singleton	ELCS	Spontaneous labour	EMCS	Term
Laura (009)	Third trimester	Primip	Singleton	Vaginal	Spontaneous labour	Vaginal	Term
Louise (011)	Third trimester	Primip	Singleton	Vaginal	Spontaneous labour	Vaginal	Term
Tracey (012)	Third trimester	Primip	Singleton	Vaginal	C-section	EMCS	Term
Sian (013)	Third trimester	Primip	Singleton	Vaginal	Induction of labour	Ventouse	Term
Jenny (016)	Third trimester	Primip	Singleton	Ambivalent/vaginal	Induction of labour	Forceps	Term
Claire (001)	Third trimester	Multip	Multiples (twins)	Vaginal	–	–	–
Shelley (003)	Second trimester	Primip	Singleton	Vaginal	–	–	–

Cat, category; C-section, caesarean section; ELCS, elective caesarean section; EMCS, emergency caesarean section; Multip, multiparous; Primip, primiparous.

**Figure 1:**
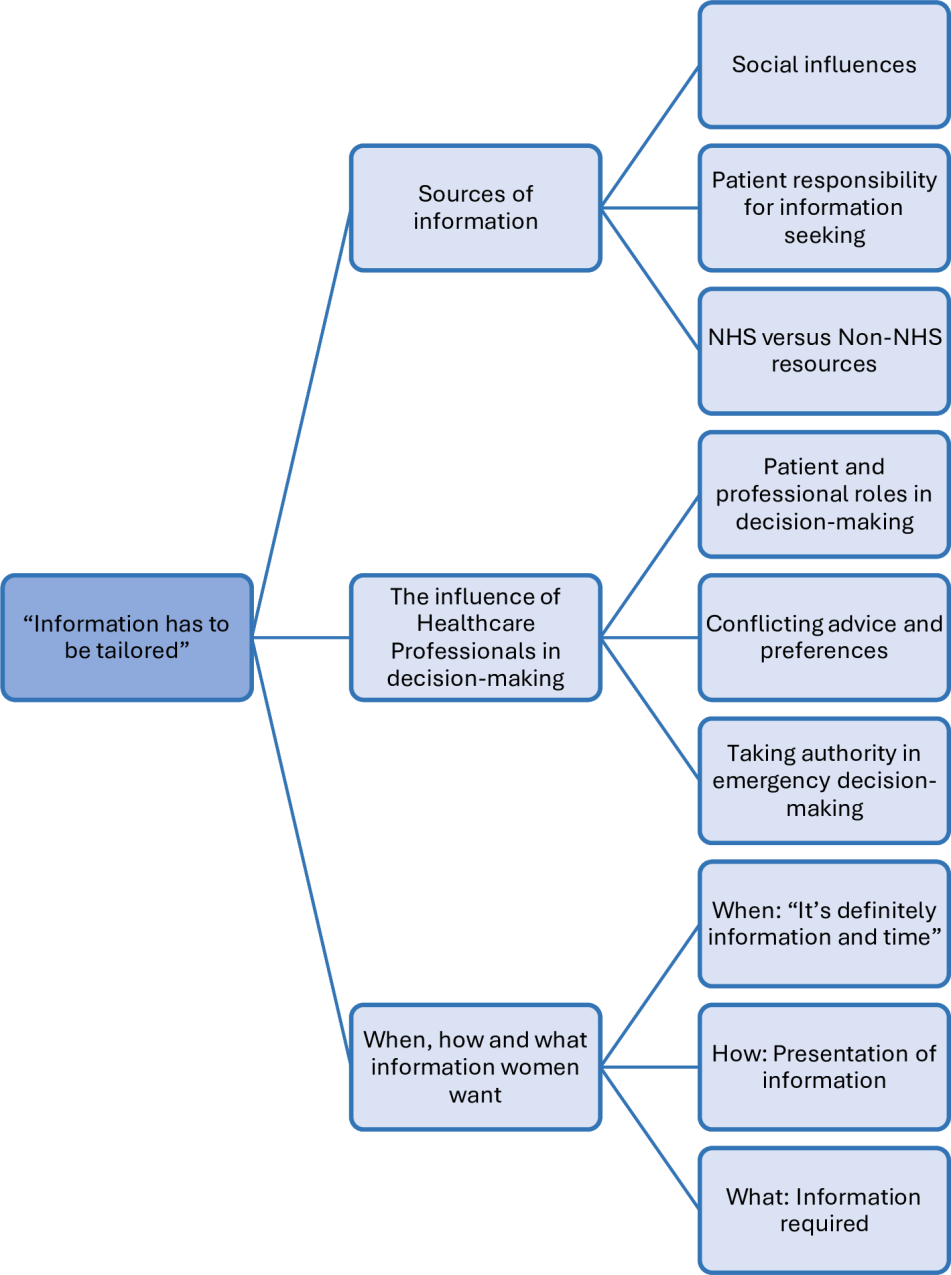
Themes and subthemes. NHS, National Health Service.

**Figure 2:**
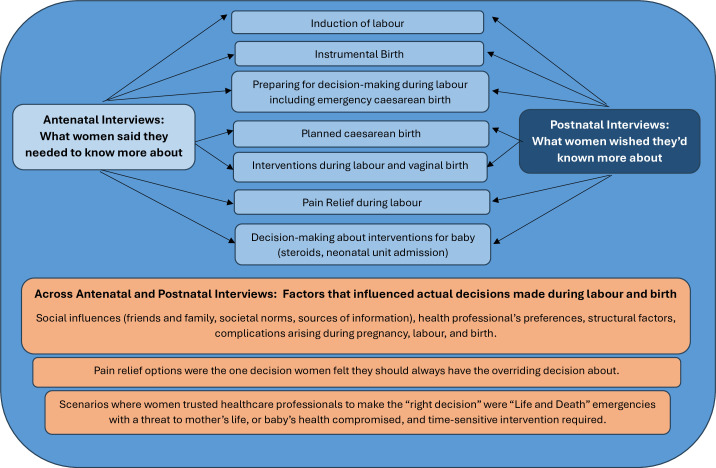
Overview of what women said they needed to know (antenatally), wish they had known (postnatally) and what influenced (actual) decisions made during pregnancy, labour and birth.

Figure 1 outlines the themes and subthemes. Three main themes were generated: Theme 1, *sources of information*; Theme 2, *the influence of healthcare professionals in decision-making*; and Theme 3, *when, how and what information women want*. The overarching theme ‘Information has to be tailored’ encapsulates the value women place on SDM when asked antenatally and postnatally. Figure 2 summarises what women wanted to know and wished they had known. Women were consistent in the information they wanted to know. Figure 2 also shows women trusted HCPs to make ‘the right decision’ in ‘life and death’ emergencies. Women’s views about factors that influence decisions did not change. Quotations from individual participants supporting each theme and subtheme are presented in table 2.

**Table 2:** Cross-case findings: themes and subthemes with illustrative paired interview data by participant

	Theme	Subtheme	Antenatal and postnatal perspectives
Decision-making ‘Information has to be tailored’ (012PN)	Theme 1: sources of information	Social influences	‘My family all feel strongly about natural birth, and my partner’s family have a very medicalised view of birth’. (012AN)
			‘My partner was involved, we had time to go away and talk to friends and family who had been through similar things’. (O12PN)
		Patient responsibility for information seeking	‘I still probably need to do a bit more reading and research’. (002AN)
			‘I wish that I had asked more about caesarean at that point, and that is I think my own fault’. (002PN)
		NHS vs non-NHS resources	‘I do tend to follow NHS to be honest because I think it’s still the best source of information’. (011AN)
			‘If it’s a professional saying it, seen hundreds of births, tons of births, you might take it with a bit more weight’. (011PN)
	Theme 2: the influence of healthcare professionals in decision-making	Patient and professional roles in decision-making	‘So they can offer their opinion, but it is very much a choice…it’s down to us making the decision’. (013AN)
			‘Yeah, I am pretty angry about the whole thing…yeah nothing on my birth choice [plan] has happened’. (013PN)
		Conflicting advice and preferences	‘You probably won’t need it [epidural), you are young, you are fit, you are healthy’ and that was really the extent of the information’. (002AN)
			‘I think it’s just the disconnect between I think I felt like I had to be really pushy and really advocate for what was happening next’. (002PN)
		Taking authority in emergency decision-making	‘Ultimately when the life of the mother or the infant is at risk, decisions have to be made [by HCPs]’. (005AN)
			‘The decision became let’s try and make sure the outcome for these babies is as good as possible, and that therefore requires us to move very quickly through a series of decisions…and fortunately I was on-board with all of those’. (005PN)
	Theme 3: when, how and what information women want	When: ‘It’s definitely information and time’	‘If you don’t understand what they are asking you…you can have the time to look at it and think yeah okay, or no I would rather wait another half an hour’. (008AN)
			‘You don’t really have time to be like let me have ten minutes to discuss this, it’s just like we need to go’. (008PN)
		How: presentation of information	‘…if you know that you’re going to be having a caesarean section for example, you don’t need to sit through lots of information about vaginal deliveries because it’s just not going to be relevant for you’. (009AN)
			‘…it’s better to have face-to-face discussion so that the responses that you want can be obviously more tailored to the questions that you’re asking’. (009PN)
		What: information required	‘…you should also be aware that there are these risks of doing it [induction] like an increased chance of further interventions, an increased chance of ending up having a caesarean section’. (012AN)
			‘They didn’t tell us the risks just as we were going into theatre, they told us the risks long before, so things like this could increase the risk of stillbirth in the future…we needed to hear to make a good decision’. (012PN)

AN, antenatal; NHS, National Health Service; PN, postnatal.

The analysis revealed similarities within both antenatal and postnatal participant groups, as well as across individual interviews. Consequently, the three themes and nine subthemes include data from antenatal and postnatal interviews. While women’s perspectives on factors influencing decision-making remained consistent across antenatal and postnatal interviews, three subthemes do include lived experiences of labour and birth more than others (ie, taking authority in emergency decision-making). This reflects how some concepts were more thoroughly explored by participants in postnatal interviews. There were also nuances in postnatal interviews relating to how the potential negative influences from friends and family’s birth experiences (antenatally) played out in reflections of their own actual birth experiences, how HCPs’ preferences during labour affected decision-making and the lack of time to consider options during actual labour affected birth experiences. Two women reported differences between birth preferences and actual experience. One changed her mind about the induction of labour (002); and one would have preferred an elective caesarean section (013). Neither felt informed enough to make that decision antenatally.

All interviews took place between October 2021 and May 2022 during the NHS COVID-19 recovery period. COVID-19 was spontaneously mentioned in 7/22 interviews, but there was no clear pattern to responses concerning decision-making. One woman felt there was a lack of antenatal education information leaflets, while another woman thought the shift to online antenatal education information was a good thing and very helpful. Two women talked about their experience of having COVID-19 during pregnancy. Another woman thought COVID-19 adjustments did not affect her care at all in comparison to her friend’s birth experience during the first lockdown (March 2020). Two women felt their care was negatively affected during pregnancy. These women said they could not access a dating scan or external cephalic conversion. One further woman mentioned she wanted to avoid induction of labour because of restrictions on birth partner visiting. It is unclear if this was the Trust policy at the time of her birth or based on other women’s accounts from the first few months of the pandemic response.

In the following sections, each theme and subtheme are discussed in turn, including supporting selected quotations from the antenatal and postnatal interviews. Participant number and timepoint (antenatal (AN) and postnatal (PN)) are parenthesised alongside quotations. Additional quotations to support each theme are available (online supplemental tables S5–S7).

### Theme 1: sources of information

We identified important differences in the sources of information women access, use and value. Where and how women sought antenatal information seemed to influence their contributions to decision-making for labour and birth.

#### Subtheme 1.1: social influences

The experiences and opinions of friends and family shaped the decisions women made for labour and birth. Most women felt that friends and family offered valuable and trusted information to assist them with decision-making. Women said that advice from others who had positive birth experiences provided comfort when facing decisions about intrapartum interventions. However, it was other women’s stories of negative experiences that influenced actual decisions made, ‘I have heard people who have been induced (…) found it really tough, so that’s why I think I would probably want an epidural’ (016AN). Additionally, women recognised how reliance on friends’ and family’s opinions and experiences can narrow options for labour and birth, ‘(…) negativities I guess around caesarean option, I almost discounted it without really thinking about it’ (002PN). In antenatal interviews, women reported that other women often described difficult births, ‘a lot of my friends have had babies and said it’s almost like a competition who had the worst birth’ (008AN). Postnatally, women compared what they had heard about difficult birth experiences from friends and family with their own experiences, suggesting that their experiences were not so bad, ‘If anything it was like friends and family putting me off it [stretch and sweep] saying (…) it would be uncomfortable (…) but actually it was fine’ (009PN).

Societal norms influenced how women interpreted available information. During antenatal and postnatal interviews, women reported stigma associated with caesarean section, induction of labour, assisted vaginal birth and pain relief options, and how culture influenced their thoughts about the decisions to be made. As encapsulated in the following quote, some women held the belief that, ‘(…) in society there is almost this sense of you can do it as a woman’ (005AN). Alongside this, particularly postnatally, women highlighted that societal stigmas are unrealistic and unhelpful to women’s birth experience, ‘There’s a bit of a stigmatism[sic] around having an epidural, you feel like you’re cheating or whatever, but actually it was the nicest thing in the entire world’(008PN).

#### Subtheme 1.2: patient responsibility for information seeking

Women felt that they had to take responsibility for obtaining information about labour and birth. One participant described this particularly when asked about information they wish to receive about birth interventions before labour, ‘I think sometimes you have got to take a bit of responsibility yourself. If you want to understand it, you need to go and read it’ (008AN). Other women described taking responsibility to seek out information for labour and birth, either due to insufficient antenatal information provided or because they wanted more details on specific options, ‘I haven’t been given any information, it’s only what I have researched myself’ (003AN). Some women were proactive in sourcing information from websites, books, apps and private antenatal classes, including the National Childbirth Trust (NCT), ‘I got information because I went to and paid for NCT (…) I wouldn’t have had any information unless I sought it out from community midwives’ (002 PN). Other women felt more information should be provided by the NHS and lacked knowledge in preparation for labour and birth. One participant expressed the responsibility not only to seek their own antenatal information, but to also initiate SDM during labour and delivery, attributing this to a lack of communication around specific choices, ‘I felt some of the decisions were more needed to be initiated more by myself as opposed to being raised by the midwives’ (016PN).

#### Subtheme 1.3: National Health Service (NHS) versus non-NHS resources

During antenatal and postnatal interviews, women described how they sought information from a variety of NHS and non-NHS resources with mixed views on which were best and why. Many women found NHS information interfaces lacked personal relevance, which at times deterred them from engaging with it, ‘The information in the NHS website I find is very generic (…)’ (001AN) and ‘a lot of the NHS resources and website and everything just looks super corporate (…)’ (004PN). Nevertheless, most women perceived that NHS information was the most reliable source of information available and therefore used it despite these issues, ‘I do tend to follow NHS to be honest because that’s…I think it’s still the best source of information (…)’ (011AN) and ‘I personally try not to read things online unless they are on the NHS website (…) only use the NHS as a source of truth’ (008AN).

Women also sought information from non-NHS sources such as private antenatal classes or non-NHS apps. However, these resources were perceived as more restrictive and in support of specific birth choices, ‘I felt like the [private] antenatal class was a bit too biased towards hypnobirthing, and the promotion of natural birth’ (013AN), a different private antenatal class provider, ‘was really quite negative about caesarean, (…) and six out of seven of our group ended up having caesareans’ (002 PN). Some women therefore preferred to seek NHS information over these classes. One participant also expressed concern over contradictions in information provided by NHS vs non-NHS resources, ‘(…) the information from [private class] and NHS was not quite marrying up’ (004PN). NHS and non-NHS were both valued, but inconsistencies caused concern.

### Theme 2: the influence of healthcare professionals in decision-making

The second theme among antenatal and postnatal interviews was the influence of HCPs in decision-making for labour and birth. Participants described collaborative decision-making alongside the HCP, how the preferences of the HCP affected choices available, and the authority of the HCP over decisions in an emergency.

#### Subtheme 2.1: patient and professional roles in decision-making

Almost all participants understood SDM as the process of making choices together: ‘a balance with the woman deciding with the professional’ (013AN). Antenatally, women wanted involvement in most decisions with their HCPs, ‘For me, yeah, I generally want to participate in everything, that’s my assumption’ (004AN). A few women thought decision-making authority should lie with the birthing person, ‘I think the midwife and the doctor would tell you the information that you need to know (…) but then ultimately the decision is yours’ (007AN). Postnatally, women’s views and experiences encapsulated how the balance of SDM is contextual and dependent on the nature of the decision.

During labour, the pressures of the busy hospital environment limited HCP capacity to support DM as reported by this woman: ‘the four midwives who were all wonderful but just too overwhelmed to ever have a discussion that could constitute shared decision-making’ (002PN). Some women did feel they were supported, particularly in decision-making about episiotomy and instrumental birth, despite the fact that decisions were too quick to be considered shared: ‘I remember her asking me if it was okay that’s what they were going to do, is it okay, and I said yes (…) so it was shared in a sense, but it was also quite a quick decision’. (016PN). Even when there was a time to reflect on a decision and decisions were being made under less time pressure (induction, epidurals), not all participants felt they had autonomy around/had participated in shared decision-making, ‘A lot of the decisions were taken from me…I handed my body over’ (005PN) and ‘I don’t believe that bit was as much of a shared decision as it could have been’ (008PN). This left one participant ‘angry’ (013PN) as her birth choices did not occur as planned.

Despite this, five women detailed positive birth experiences, and three women discussed experiences that were neither positive nor negative, but they felt supported by the HCPs in making decisions, ‘It was shared, but the decisions weren’t anything different from what I wanted (009PN). Some women in some circumstances actually preferred to not be the ultimate decision maker, ‘It made me feel safer to not make that decision in a way’ (012PN).

#### Subtheme 2.2: conflicting advice and preferences

Some women perceived how HCPs provide information reflecting the HCPs preferences, ‘I worry that the information that I am being given is presented from a perspective of what actually makes healthcare professionals more comfortable’ (001AN). One woman discussed how antenatal advice provided by HCPs was ‘a little pushy around natural birth, very dismissive of any other pain relief options, or discussion around that, which was a little tricky’ (002AN). Postnatally, women reflected on how this sometimes made decisions difficult as they sometimes felt the need to advocate for certain choices in the intrapartum period, ‘I felt like I was already having to almost persuade them that this is a change of plan’ (002PN). Both antenatally and postnatally, women therefore discussed how the preferences of the HCP caused them to feel a lack of control and autonomy, leading to some outcomes that did not meet their expectations, ‘I felt really pushed and I ended up agreeing to an induction in the end’ (012 AN) and ‘So then they said that they wanted me to have the drip, which was not really what I wanted’ (013PN).

Some participants also highlighted that different HCPs occasionally offered differing advice and opinions on choices for labour and birth, which subsequently made decision-making challenging, ‘So I guess there was conflict, not conflict, but there’s definitely different opinions depending on who you spoke to, I felt like through the journey’ (016 PN). This was often attributed to their role, seniority and experience grade, ‘different doctors are different grades as well…Then you speak to your midwives who…have a very different perspective altogether’ (001AN). Inconsistency was a common reflection among participants antenatally and postnatally, suggesting the need for better standardisation, ‘It felt like the information from the two sources didn’t exactly marry up, like they hadn’t come from the same perspective’ (004PN).

#### Subtheme 2.3: taking authority in emergency decision-making

Women were asked if there were any scenarios in which SDM with HCP would not apply. Antenatally, there was consensus that: ‘…if there was a situation where I was unable to make the best decision for me at that time, then I would want [the HCP] to take that decision out of my hands’ (003AN). Between women, while there were different definitions of an emergency, the HCP always held the key to communicating information and decision-making. Emergencies were variously described as a threat to their life, compromised health of the baby, or when time-sensitive decisions were necessary, ‘I was expectant that the decision about the labour or the birth was going to be dictated to me by the consultant when he said, ‘Okay so the placenta now is not functioning, we need to get the baby out’ (005PN). Women acknowledged how the pain of labour made communicating their preferences and their role in DM a challenge. Although most women were happy for a HCP to take over decision-making in an emergency, both antenatally and postnatally women articulated concerns over their lack of control. Most women deliberated about how decisions should be communicated to them or their birthing partner during emergencies, ‘They should tell me or my partner what they are doing and say why they are doing it, but I don’t think it should be a conversation as such’ (011AN). Two participants wanted potential decisions to be made during an emergency to be discussed with them in advance to retain their role in the decision-making process, ‘I would expect that I had been asked to make the decision beforehand and that they may remind me of that.’ (012AN).

### Theme 3: when, how and what information women want

Theme 3 reports the practicalities of better equipping women with antenatal information about their options for labour and birth. In particular, the timing of information, presentation and content.

#### Subtheme 3.3: when: ‘it’s definitely information and time’

In both antenatal and postnatal interviews, the timepoint that information was given and how long women had to process it before being involved in DM was important. ‘It’s definitely information and time, so giving you as much information as possible, and then giving you the time to process it and make a decision’ (008AN). Women who received limited antenatal information expressed hope that more information would become accessible over time, ‘ I thought, oh well, I will potentially have an appointment later down the line where I can talk about this’ (002AN). Another woman assumed information would be provided at the right time, ‘So no, I haven’t got the information yet and I am not worried about that at all’ (005AN). Postnatally, some women felt that having all the information prior to labour positively affected their birth experience; hence, all information should be provided before the intrapartum period, ‘…what I found most helpful for me was having all of that information beforehand’ (009PN). In both antenatal and postnatal interviews, many women referred to a lack of time to process and consider options for labour and birth, ‘they don’t have a lot of time with you and I needed to take that fact on board’ (001AN) and ‘I was rushing to get all my questions out really quickly, and not really taking in the answers’’ (002 PN). Postnatally, women reflected on how they were also left unable to ask questions during labour and birth due to time constraints within the NHS, ‘obviously they’re busy, they were so busy, there was no one actually available to speak to us directly (…)’ (013PN). Over half of the participants stated that they still had a positive birth experience despite the effects of a lack of time, ‘I feel like I sound like I have been quite negative and actually it was overall a very positive experience (…)’ (002 PN). Only one participant felt they had adequate time to consider options in labour which contributed to their positive birth experience, ‘We had loads of time and space to make the decision, people weren’t rushing us at all’ (012PN).

#### Subtheme 3.2: how—presentation of information

Throughout the antenatal and postnatal interviews, women discussed how the presentation of information can influence decision-making. First, women explored the differences between verbal vs written information. Some women preferred access to information verbally over written information to aid further discussions and questions, ‘I think generally having someone there to talk to is really good (…) I think I wouldn’t want to be given a piece of paper’ (013AN) and ‘written information it’s quite static information, you almost need to have the opportunity to ask questions if you don’t understand something that’s written’ (009PN). Some women preferred exclusively written information so that they could have time to review and process it, ‘I felt like if I could read something and process it in my own time, that would have helped. But it was just spoken to me about’ (007PN). Some women described how having verbal information first, and then written information to supplement this would be of benefit, ‘Definitely a conversation to begin with (…) and then almost have a really high level sheet, a piece of paper or a leaflet or something afterwards that you can refer back to’ (008AN). The majority of women, however, expressed the view that a hybrid of written and verbal methods is the most effective approach for presenting antenatal information. Antenatally, women also proposed a visual decision-making tool as a simple visual way of providing information to assist them in decision-making for labour and birth, ‘For me personally, I would imagine things like information sheets coupled with a decision-making tool could be useful’ (005AN). The usefulness of further information tools such as apps, websites and books was also considered, and many women discussed the helpfulness of online forums discussing other women’s birthing experiences to aid decision-making.

Both antenatally and postnatally, there was also emphasis on the significance of tailoring and personalising the presentation of information depending on the preference of the individual, ‘I think pain relief should be something on a handout, because that’s choice’ (011AN) and ‘I think for some women that’s quite a major surgery (caesarean section) and so I think it’s about tailoring the advice I guess to what people are expecting’ (009PN). Hence, it was discussed that information should be presented considering both the importance of the decision medically and the relevance of the decision to the individual, ‘I suppose it all depends on how big the decision is, and what the decision is about’ (013AN).

#### Subtheme 3.3: what—information required

It was apparent that women who felt that they had adequate information, whether from a HCP or other information source, still lacked knowledge in some key areas surrounding labour and birth. Women were often knowledgeable about specific singular interventions, yet discussed other specific antenatal information that they felt they required to assist them with decision-making. Women wanted more information about induction of labour, instrumental birth, pain relief, pelvic examination, foetal monitoring, anti-sickness injections and the immediate postnatal experience. During antenatal interviews, women also reported that they wanted additional information about caesarean section, ‘Because I feel like opting for a C-section, if I can manage a vaginal delivery is a big con, just because I feel like the recovery will be harder, and I do wonder whether I have no evidence on this (…)’ (001AN) and ‘I certainly had more information about a stretch and sweep than I did about a caesarean’ (002 PN). Four women had an emergency caesarean birth but had received no information about what to expect. Some women felt that they required every available detail of information to assist them with decision-making, for example, details of the pros and cons of specific options, ‘they probably haven’t really done much of the pros and cons on C-sections or vaginal delivery with me’ (001AN). One participant felt that information concerning the pros and cons of every option may not facilitate decision-making for some individuals, ‘Only talking about risks without talking about benefits makes me feel threatened (…) that’s harder then to make decisions’ (012AN). Therefore, there was concern that providing extensive information could complicate decision-making; hence, some women suggested the need to tailor information to the individual, ‘It can almost give too much information, and so it’s better to have face-to-face discussion so that the responses that you want can be obviously more tailored to the questions that you’re asking’ (009PN).

## Discussion

In this qualitative antenatal-postnatal paired interview study on decision-making for labour and birth, we found similarities and differences in women’s views about decision-making and information requirements when they were interviewed before and after delivery. The lived experience of birth revealed the potential negative influences of friends, family and society on antenatal information provision; the influence of healthcare professionals’ preferences; and the limited time to consider options in labour on decision-making. Women valued information about labour and birth, and participating in decision-making, but at the same time, they recognised the process can be compromised by emergencies, HCPs’ workload and preferences and structural factors. Participants felt they were sometimes responsible for sourcing information about birth and identified inconsistencies in available information that required navigation. Five participants described positive birth experiences despite antenatal concerns over a lack of information on their options for labour and birth. Only one participant felt they had a negative birth experience. Too little information, too late and with too little time for discussion in NHS maternity care contexts were barriers to decision-making. Women wanted to know more about labour, induction of labour and caesarean birth (planned and emergency). We found women wanted access to reliable, timely information and for information to be tailored to their needs.

### What this study adds in relation to existing research

This study adds new qualitative evidence from repeat interviews with the same women that supports professional calls for standardised sources of reliable and comprehensive information about options for labour and birth.^49^ Consistent with previous research, we found that while women gather or receive information about labour and birth from an array of sources, this does not necessarily translate to consistent, accessible or timely information that women want to inform safe and personalised care during labour and birth. Women are often exposed to copious amounts of information available from friends, family, social networks and media. The provider of information for labour and birth has been found to introduce prejudices and personal beliefs that can both positively or negatively shape experiences and outcomes for labour and birth.^22 50 51^ Although social support can support a positive antenatal experience,^52^ particularly cultural background has been linked with negative birth and decision-making experiences.^53^ Some women therefore narrowed their knowledge of birth options, for example, caesarean birth, based on these prejudices and social views, meaning they felt they were not provided with adequate information about other options. Antenatal classes have been proven to reduce maternal stress and mental health outcomes, yet also to reflect societal norms.^25^ Women often rely on the experiences of others and trust their ability to seek out personalised information, making it challenging to standardise perspectives. Ensuring women, use standardised sources of information such as core information sets would provide balance and could reduce these biases.^54 55^

In this study, repeat interviews provided new insights into the interplay of information, communication and interactions surrounding shared decision-making as perceived by women before and after their experience of birth. Investigating women’s perceptions of decision-making during pregnancy and the postnatal period is more methodologically rigorous than exploration at a single time point. Post-partum quantitative and qualitative studies investigating women’s preferences, expectations and experiences of birth have been criticised as open to recall bias in successive systematic reviews.^16 26 56^ A 2021 systematic review of quantitative measures of the mismatch between expectations and experiences included only 11 prospective studies.^16^ As a method, the advantages of repeat interviews include that they can provide rich, contextualised and dynamic accounts of evolving and complex phenomena.^57^ This study adds new insights into the similarities and differences between women’s birth expectations and experiences of birth. The strength of similarities between antenatal and postnatal paired interviews in this study was unanticipated. Women’s priorities for information provided and views about influences on decision-making during pregnancy and after birth did not change. This was only evident because of the antenatal-postnatal paired interview design. The differences between antenatal and postnatal paired interview findings resonate with previous longitudinal studies that found experiential, embodied knowledge is gained by giving birth.^58 59^ The nuances in the postnatal findings could be addressed by the inclusion of women’s stories in decision-making information, alongside recognition that, and preparation for, birth does not always go to plan. This study suggests that women should be prepared for decision-making to be a dynamic process, whereby the impact of social influences, time constraints and the role of healthcare professionals will evolve. In common with the conclusion of a systematic review of 13 qualitative and 19 quantitative studies of pain relief during labour, this study shows women should make decisions based on what they would like to happen, while having an understanding of what might happen.26 One study of women’s online chat forums suggests women commonly acknowledge the need to go with the flow.^60^ Existing research shows pandemic-driven changes impacted NHS maternity and neonatal service delivery, user experience and staff well-being in different ways, at different times.^61^ While not the focus of this study, COVID-19 was spontaneously mentioned in 7/22 interviews, but not consistently about decision-making.

### Implications for clinicians and policymakers

Addressing the need for information does not always require an increase in the quantity of information, but improving how available information is tailored to assist in decision-making practices.^62^ However, there is an inherent challenge that it is uncertain antenatally how labour and birth will evolve. The role of the HCP can be seen as the catalyst for bridging information between external sources, the needs of the patient and what NHS Trusts offer.^21 54^ Our findings resonate with existing qualitative systematic reviews that highlight the key ingredients for a positive birth experience,^13^ the role of HCPs and organisations in SDM interactions, and women’s needs for emotional support and dialogue with HCPs alongside information about birth.^63^,^65^ Our companion study on maternity HCPs’ perspectives on decision-making explores the unique challenges and complexities of a HCP’s role in respecting patient autonomy, adequate communication and providing the patient with emotional support.^21^ Women value the knowledge and expertise of the HCP and sometimes express the need for assistance with choices, especially in emergencies.^21^ Yet as the *Montgomery vs Lanarkshire* case highlights, a HCP should not limit choices, and their personal preferences should not interfere with the decisional autonomy of the birthing person.^19 20^ HCPs have a legal obligation to provide necessary information for decision-making, yet the extent of their involvement should be decided by the woman. Although these barriers are structural rather than relating to the individual practitioner, HCPs should acknowledge that some women may prefer to have more involvement in decisions than others. ‘Tailoring’ an individual’s preferences on decisional autonomy early in the antenatal period would be a way to reduce potential barriers that a HCP may pose. In the present study, women identified a lack of time to discuss options and consider choices together with their HCPs, which is difficult to resolve due to increasing demand for the NHS facilities and staffing requirements.^66^ It is therefore appropriate to assess the best way to mitigate the effects of timing of information on decision-making by providing women with adequate and personalised information.^50^ ‘How’ women should be provided with this information and ‘what’ information they should be provided with is most important to address. There are several solutions. These include greater use of decision aids,^67^ birth plans^68^ or structured counselling using core information sets.^5569^,^71^

### Unanswered questions and future research

The overall goal for aiding improvement in decision-making practices for labour and birth is to facilitate more positive and fulfilled birth experiences and meet legal requirements. A positive birth experience encompasses personal, cultural and emotional beliefs alongside clinical and psychological support.^51^ Another of our studies on the postnatal perspectives of decision-making for intrapartum interventions also highlights the inconsistency of information they have received about both mode of birth and induction of labour, despite SDM being prioritised by NHS improvement.^31 36^ This impacted women’s birth experiences overall.^36^ Subsequent research on improving and tailoring antenatal information provision should reflect each woman’s evolving experiences and not be limited to a specific time point.^16^ Women must also have access to consistent, accurate information that ‘they want or need in a way they can understand’.^72^ The development of further core information sets can be used to aid more detailed discussions, along with decision-making tools including infographics that are personalised for individuals.^55^ Further research investigating how women reconcile conflicting information is also warranted given the myriad of information available.

### Strengths and weaknesses of this study

The antenatal and postnatal paired interview design facilitated the exploration of new data from the same women before and after birth. It did not show a shift in individuals' views about decision-making for labour and birth between interviews. We acknowledge that this may be influenced by the small sample of women in this study and the sociodemographic background of participants. Where repeat interviews have been used before in longitudinal studies of pregnancy and childbirth,^58 59^ they have highlighted incongruity between the blandness of information given and how powerfully women experience giving birth. This may explain why information about labour pain, induction of labour and caesarean section was felt to be inadequate. It is a further strength of the paired interview design that it illustrates women’s appreciation postnatally that some decisions should be made at pace and that decisions may shift, although this is not to say women did not want information before or after the event regarding emergency situations. The trustworthiness of findings is enhanced by independent analysis and the agreement of all authors on the final themes. Limitations of this study include recruitment from a single site, the inclusion of only one multiparous woman, and we did not record ethnicity or socioeconomic data. It is unclear to what extent their information and care needs were exacerbated by the COVID-19 pandemic.

## Conclusions

In this qualitative antenatal-postnatal paired interview study on decision-making for labour and birth, we found few differences in women’s views before and after giving birth. Women understand decision-making during labour and birth is a dynamic process. Women can struggle with the volume, quality and timing of information available. In busy maternity settings, the challenge is to better equip women with the information they want, and health professionals with the information they need to provide for personalised care and shared decision-making. Antenatal interventions that warrant further research include decision aids, birth plans and structured counselling using core information sets. When addressing ways to improve antenatal information provision, insights from both antenatal and postnatal perspectives can help.

## Supplementary material

10.1136/bmjopen-2024-096171 online supplemental file 1

10.1136/bmjopen-2024-096171 online supplemental file 2

10.1136/bmjopen-2024-096171 online supplemental file 3

10.1136/bmjopen-2024-096171 online supplemental file 4

10.1136/bmjopen-2024-096171 online supplemental file 5

## Data Availability

All data relevant to the study are included in the article or uploaded as supplementary information.
