# Supplementary material for: Women’s perspectives of decision-making for labour and birth: a qualitative antenatal-postnatal paired interview study
Source: BMJ Open. 2025 Jun 4;15(6):e096171. doi: 10.1136/bmjopen-2024-096171 (PMC12142090; doi:10.1136/bmjopen-2024-096171)
Supplement: online supplemental file 1 [file bmjopen-15-6-s001.docx]

| **No.** | **Topic** | **Item** | **Page Number** |
| --- | --- | --- | --- |
| **Title and abstract** | | |  |
| S1 | **Title** | Identifies the study as a qualitative antenatal-postnatal paired interview study. | 1 |
| S2 | **Abstract** | Summary of key elements including data collection and data analysis. | 2-3 |
| **Introduction** | | |  |
| S3 | **Problem formulation** | Description and significance of shared decision-making in medicine and maternity. | 4-5 |
| S4 | **Purpose or research question** | This study aims to identify and compare antenatal and postnatal perceptions of information needs and decision-making for labour and birth by analysis of paired interview data from the same women. | 5 |
| **Methods** | | |  |
| S5 | **Qualitative approach and research paradigm** | Interpretivist approach to better understand women/birthing people's views and experiences | 6 |
| S6 | **Researcher characteristics and reflexivity** | Paragraph included about research team and reflexive accounting. To address bias arising from personal beliefs about shared decision-making final themes and sub-themes generated were agreed upon by the whole research team. | 7 |
| S7 | **Context** | Single NHS Trust in the South-West of England, with approximately 6,000 deliveries per annum. | 6 |
| S8 | **Sampling strategy** | Purposive. For representation across a range of socio-demographic backgrounds and breadth of birth experiences, we approached both low and high-risk antenatal women in their first or subsequent pregnancy. Potential participants received study information from community midwives, in antenatal clinics and on antenatal wards, which included a contact for the study team who then screened and arranged an individual interview from 12 weeks’ gestation. | 6 |
| S9 | **Ethical issues pertaining to human subjects** | This research was approved by the Health Research Authority (HRA) and an NHS Research Ethics Committee (Reference number 20/SW/0035). | 20 |
| S10 | **Data collection methods** | Twelve antenatal and ten postnatal interviews. | 6 |
| S11 | **Data collection instruments and technologies** | Semi-structured topic guide exploring decision-making for labour and birth, consent, knowledge of birthing interventions and options. | 7 |
| S12 | **Units of study** | Pregnant and postnatal women. | 6 |
| S13 | **Data processing** | Interviews were audio recorded using an encrypted device and transcribed verbatim. All antenatal and postnatal interview data were coded using Microsoft Excel. | 7 |
| S14 | **Data analysis** | Braun and Clarke’s six-stage method of thematic analysis was used. All authors independently agreed that sufficient data was achieved to claim saturation. | 7 |
| S15 | **Techniques to enhance trustworthiness** | For trustworthiness, analysis was carried out independently and then reviewed collaboratively at each stage (EW, AM, CK). | 7 |
| **Results/findings** | | |  |
| S16 | **Synthesis and interpretation** | Themes and subthemes are outlined in results section and shown in figure 1. | 8 |
| S17 | **Links to empirical data** | Quotes integrated within results and in Table 2. | 7-16 |
| **Discussion** | | |  |
| S18 | **Integration with prior work, implications, transferability, and contribution(s) to the field** | Discussion section has paragraphs detailing what this study adds and findings in relations to other studies. | 16-18 |
| S19 | **Limitations** | A paragraph in the Discussion section. | 18 |
| **Other** | | |  |
| S20 | **Conflicts of interest** | None declared. | 20 |
| S21 | **Funding** | This work was supported by a David Telling Trust award. | 19 |

Reference 36: Brien B, Harris I, Beckman T, Reed D, Cook D. Standards for reporting qualitative research. Acad Med. 2014;89(9):1245-51.
