# Supplementary material for: Women’s perspectives of decision-making for labour and birth: a qualitative antenatal-postnatal paired interview study
Source: BMJ Open. 2025 Jun 4;15(6):e096171. doi: 10.1136/bmjopen-2024-096171 (PMC12142090; doi:10.1136/bmjopen-2024-096171)
Supplement: online supplemental file 2 [file bmjopen-15-6-s002.docx]

**Supplementary Table 2**

**Table S2**: *Inclusion & Exclusion Criteria*

| **Inclusion Criteria** | **Exclusion criteria** |
| --- | --- |
| Women who are going to receive intrapartum care. | Women who have not or are not about to receive intrapartum care for example women who have had a first trimester miscarriage. |
| Women who are aged 18 and over | Women who are under the age of 18 |
| Pregnancy beyond 12 weeks’ gestation | Women who are not able to give informed consent to participate in the study and |
| Booked in for a delivery at the NHS Trust or attending community midwifery services in the Trust’s area. | Women who are booked for their pregnancy at another NHS trust |
| Women who are English- speaking | Women who do not speak English adequately to participate in an interview. |
