# Supplementary material for: Women’s perspectives of decision-making for labour and birth: a qualitative antenatal-postnatal paired interview study
Source: BMJ Open. 2025 Jun 4;15(6):e096171. doi: 10.1136/bmjopen-2024-096171 (PMC12142090; doi:10.1136/bmjopen-2024-096171)
Supplement: online supplemental file 3 [file bmjopen-15-6-s003.docx]

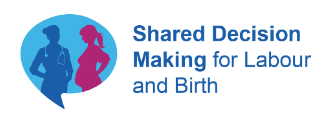


**Supplementary 3: Interview Topic Guide**

**Introduction to Antenatal interview:**

The aim of this interview is to understand your thoughts about labour and birth and how well you feel you are currently prepared to make these decisions. We would also like to hear about your thoughts on shared decision making in labour and what you and maternity staff can do to make it as good as possible.

**Topic area 1: What are you currently planning for your labour and birth?**

Prompts: Location, how, pain relief, the room, the people present

**Topic area 2: Have you thought about any interventions that may be available to you during labour and birth?**

Prompts: Vaginal examinations, blood tests, fetal monitoring, instrumental delivery, caesarean, active management of third stage, induction of labour.

**Topic area 3: Do you feel that you have enough information about these interventions?**

Prompts: Who/where do you get your information from? What did you think of it? Do you feel it was reliable? How could it be improved?

**Topic area 4: Do you think that having information about intervention before birth is a good thing?**

**Topic area 5: What are your thoughts about making decisions during labour and birth?**

Prompts: who is involved? When will they take place? How should they be recorded?

**Topic area 6: How do you think that you could be best supported to make decisions during birth?**

Prompts: decision making tools, information sheets, discussion with staff, being left alone

**Topic area 7: is there anything that we could do before labour/birth to support you in making decisions during labour and birth?**

Prompts: information leaflets / lots of facts / minimal general information/ videos

**Topic area 8: are there any areas you feel are a priority for our focus in terms of shared decision making?**

Prompts; what interventions worry you most? What interventions are you expecting? What do you feel there is least information about?

**Topic area 9: Are there any interventions that you believe should not involve shared decision making during labour?**

Prompts: do you want to participate in shared decision making? Life threatening emergencies are not usually expected to involve shared decision making – what is a life-threatening emergency to you?

**Introduction to postnatal - paired interview:**

The aim of this interview is follow up on the initial interview and see if you have any extra thoughts having given birth to your baby.

**Topic area 1: Can you tell me about your labour and birth?**

Prompts: Location, how, pain relief, the room, the people present

**Topic area 2: Did your labour and birth meet your expectations?**

Prompts: did you deliver where you planned in the way that you planned? Did you have to evolve your plans?

**Topic area 3: Did you experience any interventions?**

Prompts: vaginal examinations, episiotomy, repair of tear

**Topic area 4: Did you feel that there was a shared decision making process for the interventions?**

Prompts: did your midwife/doctor explain things to you? Did you have time to ask all the questions you wanted? Were you able to concentrate on the discussions? Would you have liked more information prior to labour?

**Topic area 5: Did you feel well equipped to make decisions about interventions?**

Prompts: Did you know the information you needed already? Had you investigated things beforehand? Was it the first time you are hearing about these interventions?

**Topic area 6: What are your thoughts about making decisions during labour and birth?**

Prompts: who is involved? When will they take place? How should they be recorded?

**Topic area 7: How do you think that you could be best supported to make decisions during birth?**

Prompts: decision making tools, information sheets, discussion with staff, being left alone

**Topic area 8: is there anything that we should do before labour/birth to support you in making decisions during labour and birth?**

Prompts: information leaflets / lots of facts / minimal general information/ videos

**Topic area 9: are there any areas you feel are a priority for our focus in terms of shared decision making?**

Prompts; what interventions worry you most? What interventions are you expecting? What do you feel there is least information about?

**Topic area 10: Are there any interventions that you believe should not involve shared decision making during labour?**

Prompts: do you want to participate in shared decision making? Life threatening emergencies are not usually expected to involve shared decision making – what is a life-threatening emergency to you?
