# Supplementary material for: Women’s perspectives of decision-making for labour and birth: a qualitative antenatal-postnatal paired interview study
Source: BMJ Open. 2025 Jun 4;15(6):e096171. doi: 10.1136/bmjopen-2024-096171 (PMC12142090; doi:10.1136/bmjopen-2024-096171)
Supplement: online supplemental file 4 [file bmjopen-15-6-s004.docx]

**Supplementary File 4: Thematic analysis approach with results.** A flow chart to show the approach to Braun and Clarke’s six-stage method of thematic analysis with a description of results at each stage (46-48). *HCP = Healthcare professional.*
