# Supplementary material for: Women’s perspectives of decision-making for labour and birth: a qualitative antenatal-postnatal paired interview study
Source: BMJ Open. 2025 Jun 4;15(6):e096171. doi: 10.1136/bmjopen-2024-096171 (PMC12142090; doi:10.1136/bmjopen-2024-096171)
Supplement: online supplemental file 5 [file bmjopen-15-6-s005.docx]

**Supplementary Material: Supplementary tables with additional supporting quotations for each Theme and Subtheme.**

*Theme 1 supporting quotations.*

| **Theme 1: Sources of information** |
| --- |
| **Subtheme 1.1: Social influences** |
| **Antenatal** |
| “I don’t know whether… I am trying to think. I think it’s probably just because my friends have put me off them, so I have just discounted them.” (001) |
| “I think I guess you take your friends’ experiences at face value, and I probably don’t interrogate that any further.” (001) |
| “I guess there is something about how information is framed, and I wonder whether there is this bias that we present in the UK around natural birth, non-interventional birth, this is the best thing, and obstetrics led intervention heavy births are not good.” (001) |
| “I have had a lot of friends who have had to have a caesarean and have actually had really lovely positive relatively chilled experiences despite the fact they aren’t planned, and quite good recoveries as well. So I don’t feel hugely daunted about that.” (002) |
| “ I think with friends I take it with a bit of a pinch of salt, they have both very different experiences to me” (002) |
| “I think because I have spoken to friends who have had induction but had it at 40 weeks or 41 weeks, and they have really positive stories, but it’s just the people who have induction at 37 or 38 weeks that seem to be like nothing happened.” (004) |
| “But I feel that in society there is almost this sense of you can do it as a woman, we’re empowered as women to be able to get through this, whereas things like the impact of perineal trauma, instrumental delivery, obstetric anal sphincter injury, I think is still a… and secondary lower urinary tract symptoms, lower bowel dysfunction/functional bowel disorder, sexual misfunction, these are still taboo.” (005) |
| “I have thought about that but only because I know that’s what my mum went through I think, and is my body going to be physically similar to my mum’s and that’s going to then happen with me and my baby, or I don’t know, are we going to be completely different?” (009) |
| “If I’m honest lots of just negative things are always said about them [forceps] so if I can avoid them then I will, but I think if it’s considered the safest route for the baby then I would go for it anyway.” (013) |
| “So yeah I would be fine to be induced, but I think I have heard people who have been induced, a couple of my friends drip… the oxytocin drip to get the contractions started, both found it really tough.” (016) |
| **Postnatal** |
| “a lot of friends and people I have met that I have chatted to, and then you end up in a scenario where you’re absolutely knackered” (002) |
| “It didn’t seem consistent, so that led me to think that there is a natural bias because they are just following rules that have been set at a local level that aren’t consistent.” (004) |
| “In hindsight I am not sure whether I would… having heard stories I am not sure whether I would have made the same decision funnily enough.” (004) |
| “I just spoke about it to friends who have had babies before and with my partner and my mum, and just got some opinions, well experiences and stuff like that, and I did a little bit of research and that helped make my decision” (007) |
| “… if you have an epidural people quite often are like you have cheated, and people say the same things about caesareans, if you have an elective caesarean then you’re not even trying, and you’re just a bit like well actually it’s my baby and this is the way I want to do it.” (008) |
| “…comparing notes with my friends and they were saying, “No [name] that doesn’t sound normal”…” (011) |
| “I probably would never have done it, but my friend typed up a birth plan, and I was like oh that’s really good, I am going to do that.” (011) |
| **Subtheme 1.2: Patient responsibility for information seeking** |
| **Antenatal** |
| “I do try and do my own research from that perspective so that I get a sense of where I feel in relation to all that information. But then there is a danger that you get that confirmation bias where you just start looking out, you start searching for the information to disconfirm their ideas or to confirm your position.” (001) |
| “But I think overall I think you just have to be able to fit with what you have read and go with what your gut feels is the right thing for you and take a little bit from here and a little bit from there and then work out what’s going to end up being best at the end of the day.” (002) |
| “I feel like that’s your responsibility is the parent or the mother or whatever the right word is to go and find some of this…” (008) |
| “I think I am one of those people where if I didn’t have enough information I would go and seek out more information…” (009) |
| “I am a bit, I don’t know, I am not blasé, I really want this baby, but sometimes I think looking into things, researching things it scares me more than it helps me…” (011) |
| “So I feel like I was quite well informed, because of all of that [antenatal class], But that was me wanting to be informed maybe.” (013) |
| **Postnatal** |
| “I think my own fault rather than lack of information or lack of being given space to do that, because…through the whole pregnancy just hadn’t ever felt like a first choice option, it was always a last resort option.” (002) |
| “When it came to the natural labour stuff I hadn’t really done as much research on that because I had no intention of doing it, but we had gone on the… I read a few hypnobirthing books, and I was doing the breathing techniques from that.” (008) |
| “I think my only recommendation is for people to make sure they read up on it in advance…” (008) |
| “Wasn’t interested at all. I didn’t even acknowledge that it could happen in an emergency. In my mind it was really clear everything else was on the table except caesarean section, so it was really interesting that it ended up being an elective caesarean section.” (012) |
| **Subtheme 1.3: NHS versus non-NHS resources** |
| **Antenatal** |
| “So I think there is a balance to be struck so that you go into it with a level of optimism, and feeling like you can do it, and I think that’s why I felt like the NHS information on epidurals falls down. It doesn’t feel very inspiring or confidence giving, which is a shame.” (001) |
| “I personally try not to read things online unless they are on the NHS website or something, only because if you read one article that says you should do X, Y and Z and then you read a different one that says something else, so I try and only use the NHS as a source of truth.” (008) |
| “Yeah, I do, because I have had… so the antenatal class that we went to was really helpful, really informative, covered a lot of that” (009) |
| “I just trust that it’s the NHS advice isn’t it… the NHS yeah I definitely rely on, and I feel confident in…”(009) |
| “I did ask them about antenatal classes, because I didn’t know where they were or where they’re offered or anything, and she sent me a link, or showed me a link and I wrote that down and took some notes, but in the end I ended up doing one myself.” (011) |
| “I think because I had NCT first… they talk through all of these things quite a lot from their perspective…and then going to NCT probably was a bit more of a medical perspective.” (013) |
| “I don’t know where they were necessarily sourced from, and I think looking back now I would go to a class led by… I would rather go to a class led by midwife.” (013) |
| **Postnatal** |
| “I think it would have made me feel better to get some of that from you rather than from a third party, because it’s like the information from [private class] and NHS not quite marrying up” (004) |
| “I thought [private class] was rubbish on that, and the support that I got from NHS was much better.” (004) |
| “I went on another course the [private] one, which is fab, and that I learnt about the interventions as well, and again like how you just described it as a by-product of going to that class, I was around people that asked more questions, which helped my understanding.” (011) |

*Theme 2 supporting quotations.*

| **Theme 2: The influence of Healthcare Professionals in decision-making** |
| --- |
| **Subtheme 2.1: Patient and professional roles in decision-making** |
| **Antenatal** |
| “I think I needed time to get my head around it, but I think it is my decision. They have given me their advice, and it was strongly in favour of an epidural, but actually I just needed time to get my head around it, and I think that was just me letting go an ideal…” (001) |
| “When it comes to immediate life and death I would say I would not need any consultation, or I would need someone telling me what was going to happen.” (004) |
| “I don’t think there is any occasion whereby they shouldn’t be involved.” (008) |
| “I think most decisions should include me. I think the only ones that wouldn’t include me would be where I am literally physically incapacitated to make a decision, and unless that’s happening then I would expect someone to include me in that process.” (009) |
| “I was very well prepared for that thanks to the community midwives, and have been happy with how that was conducted, and that’s always felt like a shared decision, I have been really well supported with it.” (012) |
| “I think you have to be informed by the consultant that something is going to happen, but at the end of the day if they say this is what we think needs to happen because of this and you don’t really have an option either way anyway.” (013) |
| “I guess maybe it’s about building that trust and building those relationships that would actually help that shared decision-making process, because if you have got that rapport you understand that your midwife is working in your best interest.” (016) |
| **Postnatal** |
| “I just trust in what I am being told, although I sound like I am contradicting myself, I don’t want to come across as sounding untrusting of the consultants, because I really do value what they have told me.” (004) |
| “Well I think my experience was in this grey area, in that a reality is that a lot of the decisions were taken from me, because my fundamental decision was I want the babies to be okay and I handed my body over” (005) |
| “I came up with a different decision with the doctors, but then when I went away and thought about it some more and looked into it some more I decided on something else.” (007) |
| “But in terms of actual decisions I guess it was a discussion between all three of us.” (009) |
| “I really don’t think there’s any situation where shared decision-making is not important.” (012) |
| “For instances if you were unable to make a decision at that time due to pain or, I don’t know, medication or something, I think yeah if it was to minimise a likely risk of harm to baby I think that would be fine for me.” (016) |
| **Subtheme 2.2: Conflicting advice and preferences** |
| **Antenatal** |
| “I would say that they clearly have their preferences… and I find that quite difficult.” (001). |
| “I was quite able to tell that’s just her take on things.” (002) |
| “I was more pumping her for questions, like what should I expect at this appointment, and how can I negotiate in a nice way with the consultant.” (004) |
| “I got the impression that my midwife as lovely as she is she isn’t an expert in gestational diabetes, and you get a lot of conflicting advice, you get people like the midwife and this anecdotal thing from the Facebook groups that suggest it will be early, and then you get the NICE guidelines.” (004) |
| “I would say he probably leant towards elective caesarean section just because of my situation in that it’s multiples and the risks associated with that… So at the moment the plan is for elective caesarean section.” (005) |
| “It’s not been consistently that, that’s the conversation I have had with doctors, whereas the birth choices team who then followed up from that conversation don’t have the same concern.” (012) |
| **Postnatal** |
| “I was having to utilise my advocacy skills lots of times during the day, and at that point I was knackered, and my partner wasn’t there for all of it.” (002) |
| “It was very different when I spoke to the consultants, they didn’t give that impression because they were coming at it from a safety getting the baby out okay perspective, and so they would be encouraging towards those things.” (004) |
| “The thing is I can’t even… so with the drugs I was almost pushing for that. So they would say, “Are you happy to have this?” I was like, “I want this,” so in that sense it was a shared decision.” (005) |
| “That’s the little bit I wish I had just listened to my body, I wish I had… that’s my only little regret, I wish I had done something differently, but I didn’t and I need to work through that at some stage.” (005) |
| “I suppose confused, yeah, and a bit unsure why you’re told one thing by one person, and another thing by another.”(013) |
| “… there were definitely differing opinions that we were given around jaundice and around giving him formula or not and things like that, and it really varied really who you spoke to.” (013) |
| **Subtheme 2.3: Taking authority in emergency decision-making** |
| **Antenatal** |
| “…if it becomes an emergency situation then it also is really out of your hands.” (001) |
| I guess if there was an emergency situation you would expect to see the doctor who would be like, ‘Right this is what we need to do now.’” (001) |
| “I know it’s tricky when things are an emergency, and probably staff will be better than others at giving you that space to have a two way discussion rather than feeling like something is being imposed on you I guess is important.” (002) |
| “I guess it slightly depends on what the intervention might be. If it is something that is more medical or emergency situation I think probably you would want to hear that from the doctor or whoever is going to be doing it I think would be the ideal scenario.” (002) |
| “if it goes completely wrong then I would really hope that you guys would just step in and do what you need to do to save us.” (004) |
| “if it was an emergency situation I would already be too anxious to be able to make an informed decision. So it’s sometimes better for someone to make it for you. Only if it was an emergency situation, and decisions had to be made quickly.” (007) |
| “…they can never force you to do anything anyway, and if it got to the point where it was an emergency and it was life or death I would rather they just did their job if that makes sense?” (008) |
| “I would still hope for some sort of information sharing.” (009) |
| “Sometimes I think I don’t want to make any decisions, I would rather be told, and if it’s life-threatening then absolutely they need to do it.” (011) |
| “I think that conversation could be had earlier for example, so there’s a possibility, and it’s an increased possibility if we have already done an intervention, that we will have to go to this option.” (012) |
| “…if it’s a real emergency then actually I think there is room for the professional taking the lead, but I know I would be fine with that but I know a lot of other people would not be fine with that.” (016) |
| **Postnatal** |
| “So we had talked about emergency caesarean, but if I was too ill to say yes crack on then of course yes crack on…” (002) |
| “I remember specifically asking her for what are the warning words that I should look out for, if they say urgent or emergency I should sit up and just follow whatever they say.”(004) |
| “So the speculum examinations, the CTG, all of those sort of interventions I was like yeah we just have to do this, and as things became more of an emergency I would have… I just trusted the guys, trusted the system.” (005) |
| “If it’s a really time-dependent decision then I think that should be stressed, and the importance of the decision, but no I think all decisions should be shared.” (007) |
| “I think there’s no point being as oh take some time to think about it, it’s not really a shared decision by that point, it’s very much like we need to get this baby out of you and we need to do it now…” (008) |
| “Life-threatening I would imagine that the patient maybe isn’t always going to make the best decision for themselves, so I would expect the medical team to take over at that point, and them just work on your basic life support, that should be professionally led at that stage.” (009) |
| “I think, calling it emergency or seeing it as an emergency that then you can’t take the time to think about, I really think that those conversations should have happened before the emergency happens.” (012). |
| “I suppose I think yeah if there was just a very high risk emergency decision that needed to be made, and professional felt like actually the safest option in that moment needing to be… would… there was a best course of action.” (016) |

*Theme 3 supporting quotations*

| **Theme 3: When how and what information women want** |
| --- |
| **Subtheme 3.1: When: “It’s definitely information and time”** |
| **Antenatal** |
| “So I think it’s the information is important and useful, but I guess maybe the timing, the placement of the information is key, and I find it quite interesting” (001) |
| “I am very conscious that NHS is under huge burdens… maybe if there was that more protected time and planned time to talk about that, that would be really helpful.” (002) |
| “If it was something that we needed to decide quickly we would just make a decision. But if it was something that wasn’t so urgent and we had time to discuss it then I would discuss it with him.” (003) |
| “I think first of all probably some time, so not having to make decisions on the spot.” (005) |
| “I think chatting to health professionals, but then also being given that time afterwards to be able to chat to my birth partner about it.” (007) |
| “so she tried to cover as much as she could, but then being given the information sheets to go away with to process in your own time and to look at when you want, and you can always refer back to them.” (007) |
| “So if somebody came in and said do you want to do A or B? You don’t have to answer there and then, you can say come back in ten minutes and I will answer the question.” (008) |
| “ I have always felt quite rushed at the appointments, but that’s because I don’t know, everyone is up against it…” (011) |
| “ I would expect that someone sits down and spends that time with me, otherwise I would be highly likely to say I am going home to think about it, and maybe that’s more risky.” (012) |
| **Postnatal** |
| “…they just didn’t have enough time to chat to you.” (002) |
| “I feel like I jumped to make a decision rather than having the time to process both sides, and I felt like if I could read something and process it in my own time that would have helped.” (007) |
| “Yeah, if you have got a little bit of time to think about it then allow people the time to think about it, whereas if there is only one option there’s not really much point.” (008) |
| “Yeah, so what I found most helpful for me was having all of that information beforehand so it’s not new information during the labour, like during that time where you’re a bit more stressed and you’re in pain, but so I went to the class, so that was class led group discussion.” (009) |
| “I understand that there will be time pressures, but that’s a conversation that should happen before…” (012) |
| “There wasn’t really time I would say to think about it, I am guessing because of the circumstance, it was she… it’s even a blur now to be honest.” (016) |
| **Subtheme 3.2: How: Presentation of information** |
| **Antenatal** |
| “I think it’s helpful to have those eventualities explained to you as well rather than just you being having to think of that or not having any prompts around that.” (002) |
| “I think if that sort of thing there had been some sort of video of what to expect as well as just… because it’s quite a practical thing that I think is quite hard to get across in writing.” (002) |
| “Probably given it written down so I could read it, and then discuss it with my partner, or the staff, whichever… whoever I feel like I need to discuss it with at the time.” (003) |
| “I would prefer to get that from you guys, because I feel like I might be looking at things that [hospital] doesn’t offer, I also might not be looking at the full list, and so it would have been nice to get a fact sheet or something like that.” (004) |
| “Just me and my partner with an information sheet would be the ideal way, because then you don’t feel pressured in that moment to just follow advice, and you can look at the information sheet.” (004) |
| “I quite like reading and processing, you can process it in your own time, and I think information leaflets was best form for me.” (007) |
| “I think the second point that you made about the talking about interventions that might happen that they are going to be more relevant for you I think is a good one, because well if you know that you’re going to be having a caesarean section for example, you don’t need to sit through lots of information about vaginal deliveries because it’s just not going to be relevant for you. So trying to tailor the information as best as possible to the mum is a good idea.” (009) |
| “I think pain relief should be something on a handout, because that’s choice, and people might change their mind, but I think that’s a choice. Induction is more medically advised.” (011) |
| “…if we were to be given a piece of paper as an additional fine, I suppose it all depends on how big the decision is, and what the decision is about, but I always think sometimes conversations are really good at ironing out any woes.” (013) |
| “I think written is always quite good, because it gives you more time to consider, and it also feels a bit more official, and also it’s good to chat through.” (013) |
| **Postnatal** |
| “So she was like, “Just let me quickly jot it down on a piece of paper so you have got it,” rather than us, I don’t know, putting in our phones or whatever… So that was just super helpful, like we carried that scrappy piece of paper around with us for a couple of days.” (002) |
| “So I was able to ask in person which was provided, but I guess actually a factsheet of what [hospital] provides could have been quite useful instead of having to wait until a consultant appointment, that might help.” (003) |
| “I would do a video, and then I would do a shorter written one that’s like a transcript.” (004) |
| ““Could this be put down in a letter, or could you give me some resources to look at and read about this?” And the answer was no…” (005) |
| “Just information sheets I think, just layout all the… what each procedure involves and the risks and benefits, just maybe bullet points and stuff like that so it’s easy to process.” (007) |
| “No, I am quite happy with just written stuff really, and I quite like to go in detail, I like to know as much as possible, but then not everyone might not want that.” (007) |
| “They were very vocal, they were very… because the problem with giving a leaflet when you’re in that much pain you aren’t going to read it anyway.” (008) |
| “Ultimately I just wasn’t going to read it, and I needed [partner] to read it… would have been probably far more inclined to have a conversation with someone.” (011) |
| “…if I was planning a caesarean section I would have probably had more specific questions rather than just thinking oh it might happen and I will just go with the flow if it does happen and I will ask stuff at the time.’’ (009) |
| “…if you don’t understand something that’s written there if you have just got written information, or even if you’re just watching a video, if it’s not interactive in any way.” (009) |
| “It can almost give too much information, and so it’s better to have face to face discussion so that the responses that you want can be obviously more tailored to the questions that you’re asking.” (009) |
| “I think discussion and time left alone, I don’t know if… depends on the situation I suppose, I don’t know if leaflets or written information would help, maybe if English is a second language, or something like that, simple written information or, I don’t know, like a flowchart or something might be helpful.” (016) |
| **Subtheme 3.3: What: Information required** |
| **Antenatal** |
| “But maybe it is useful having a bit of information about… in the run up, so if you’re talking about planned caesarean versus vaginal delivery.” (001) |
| “there’s maybe a little bit of lack of information forthcoming, but I don’t feel neglected or anything like that, I feel quite able to ask for stuff. But I think there are scenarios where people might feel a bit like that.” (002) |
| “I think in a similar vein to instrumental deliveries, it’s not spoken about loads unless you’re hearing some poor mum’s horror story…”(002) |
| “I haven’t really had much information, only the information leaflet I was given about the low PAPP-A, but as I said I have not really had any appointments to be given any additional information, so I don’t know if that comes at a later stage or whether it’s something that I should have been given.” (003) |
| “…maybe that would also be a good thing to do, is to be like this is the forceps, this is the suction machine and these are the pros and cons for both of them, so that you can weigh them up yourself, rather than having to look out for loads of different information, and compare and contrast them yourself, it to be in one place, if that makes sense.” (007) |
| “I guess you’ve got either planned or emergency…So I would want to know a little bit about each of those.” (009) |
| “I think for me in order to feel safe I need to feel not threatened, so any language that’s threatening, so only talking about risks without talking about benefits makes me feel threatened. If you say to me there’s a higher chance of your baby dying that really gets me into threat mode, and then my frontal lobes are less engaged, and that’s harder then to make decisions.” (012) |
| **Postnatal** |
| **“**So I felt like in the assessment unit there wasn’t loads of information given about that before it was just done [stretch and sweep], it was just a bit of an assumption almost that this is what we need to do.” (002) |
| “For me it would be all about induction, because the scare stories out there there’s so many of them. I would just focus on having some honest realistic case studies or just don’t call them case studies, call them stories.” (004) |
| “So I was actually when I found out that [name] wasn’t very well and had the oligohydramnios, so low liquor, interestingly there’s not a lot of information out there that’s really accessible.” (005) |
| “[more information on] what each procedure involves and the risks and benefits, just maybe bullet points and stuff like that so it’s easy to process.” (007) |
| “Not in terms of the labour itself, but I think it would be helpful to have more information about the immediate postnatal experience, and also I say postnatal but the first two weeks of what happens.” (009) |
| **“**So yeah I think induction I really think that people need more information, but I do think the hospital need to think about just to try to understand how often they are offering it, and whether it’s always actually the most sensible decision.” (012) |
| **“**I think the induction process would need more information, because the sources that you might rely on outside of clinical practice are not very… might not be very accurate.” (016) |
