# Supplementary material for: Women’s perspectives of decision-making for labour and birth: a qualitative antenatal-postnatal paired interview study
Source: BMJ Open. 2025 Jun 4;15(6):e096171. doi: 10.1136/bmjopen-2024-096171 (PMC12142090; doi:10.1136/bmjopen-2024-096171)
Supplement: Supplementary file 6 [file bmjopen-2024-096171.reviewer_comments.pdf]

PEER REVIEW HISTORY

BMJ Open publishes all reviews undertaken for accepted manuscripts. Reviewers are asked to complete a checklist review form and are provided with free text boxes to elaborate on their assessment. These free text comments are reproduced below.

ARTICLE DETAILS

Title (Provisional)

Women's perspectives of decision-making for labour and birth: A qualitative antenatal-postnatal paired interview study

Authors

White, Erin; Davies, Anna; Demetri, Andrew; McGuinness, Sheelagh; Clayton, Gemma; Fraser, Abigail; Barnfield, Sonia; Bakhbakhi, Danya; Anderson, Emma Claire; Birchenall, Katherine; Miller, Rachel; Burden, Christy; Merriel, Abi; Kingdon, Carol

---

VERSION 1 - REVIEW

**Reviewer**	**1**
**Name**	**Van Dillen, Jeroen**
**Affiliation**	**Radboud University Medical Center, Obstetrics**
**Date**	**01-Mar-2025**
**COI**	**None**

This study adds value by using repeat interviews to show how women's views on decision-making change over time. It supports existing research on inconsistent information and social influences but adds depth by highlighting shifts in perception before and after birth. However, the unique contribution could be clearer. Emphasizing how repeat interviews provide new insights and distinguishing findings from past research would strengthen the discussion

Originality - does the work add enough to what is already in the published literature? If so, what does it add?

This study adds value by using repeat interviews to show how women's views on decision-making change over time. It supports existing research on inconsistent information and social influences but adds depth by highlighting shifts in perception before and after birth. However, the unique contribution could be clearer. Emphasizing how repeat interviews provide new insights and distinguishing findings from past research would strengthen the discussion.

Importance of work to readers - does this work matter to researchers, clinicians, patients, teachers, or policymakers?

- In the section implications for clinicians and policymakers:

This section is useful for clinicians and policymakers, emphasizing the need for personalized, well-timed informationand the role of HCPs in decision-making. It highlights key barriers like time constraints but lacks clear solutions. Adding practical steps, like decision aids (e.g. birth plans?) or structured counseling, would improve its impact.

Evidence-based medicine - is the evidence presented scientifically and reliable?
The evidence is presented scientifically and is clearly described, making it reliable and well-supported.

Research question - is it clearly defined and appropriately answered?
The aims of the study are clearly described and also answered, however the specific research question is not mentioned in the paper.

Study design - is the overall design adequate?
The study design is overall adequate and clearly described. However, the number of participants is somewhat low. A detailed description of the characteristics of these participants, as well as information on diversity and representativeness, would be a valuable addition.

Participants - are they adequately described and their conditions well defined?
The inclusion and exclusion criteria for participants are clearly described, as well as the recruitment and selection process. However, a more detailed description of the participants is needed, especially considering that only 12 women were interviewed.

Also, in lines 49-54: Does low and high-risk pregnancy automatically mean different socio-demographic background? Can you explain further.

Methods - are they adequately described? Are they ethical?
- In lines 43-44, what do you mean by "interpretivist"?
- Neutralizing personal biases about SDM is clearly described in the methods section under "Research Team and Reflexivity."
- Section: Data Collection
  How was the interview guide developed, and how was it structured? If relevant topics emerged during the interviews, how were these handled in relation to the interview guide and subsequent interviews?
- Section: Data Analysis
  How were the transcripts coded? Was a specific program used (e.g., Atlas.ti)?

Results - do they answer the research question? Are they credible and well-presented?
The results do answer the research aims of this study.
- **Theme 1**: The theme effectively addresses the research aim by highlighting differences in antenatal and postnatal perceptions of information provision and decision-making. The use of direct quotes strengthens credibility, but clearer contrasts between pre- and postnatal insights would improve alignment with the research question.
- Theme 2: The theme effectively addresses the research aim by illustrating how women navigate NHS and non-NHS information sources before and after birth. The findings are well-supported by quotes, highlighting perceived reliability issues and biases. However, a clearer comparison of how these perceptions shift antenatally vs. postnatally would strengthen alignment with the research question. Additionally, further exploration of how women reconcile conflicting information could enhance the analysis.
- Theme 3: The results effectively illustrate how timing and presentation of information influence decision-making before and after birth. The participant quotes add credibility

and highlight key differences in information access, processing time, and perceived gaps. The findings provide a strong comparison between antenatal expectations and postnatal experiences. Overall, the section is well-structured, clear, and directly aligned with the research aim.

  - Subtheme 3.3: It is unclear how to determine what information each woman wants and how to tailor it to individual needs. Adding insights on methods to personalize information, such as structured consultations, decision aids (such as birth plans), or patient feedback tools, would strengthen the discussion.

Interpretation and conclusions - are they warranted by and sufficiently derived from/focused on the data? Is the message clear?

- Discussion: The discussion presents key findings well, but the comparison between antenatal and postnatal perceptions could be clearer. While some views stayed the same, the results show that birth experiences changed women's perspectives on social influences, time constraints, and the role of healthcare professionals in decision-making. The statement that there were "few differences" does not fully match the data, as some aspects of decision-making were reconsidered after birth. A clearer focus on how expectations (antenatal) compared to real experiences (postnatal) would better align with the study aim.
- Strengths and weaknesses:
  This section clearly presents the study's strengths and limitations. The paired interview design is a strong point, but the claim that no shift in views was found seems inconsistent with results showing some postnatal changes. The impact of COVID-19 on care needs is mentioned but not explored. Clarifying these points would improve accuracy.

References - are they up to date and relevant? Any omissions?
The references are up to date and relevant, with no apparent omissions.

Abstract/summary/key messages - do they reflect accurately what the paper says?

- Abstract: In the design section, consider specifying the exact timing of the first and second interviews.
- Data Collection: Include details on how participants were recruited.
- Data Analysis: Provide more information on how the thematic analysis was conducted. Did you use any specific tools or software?

Reporting standards - does the paper comply with relevant standards? If the paper is a randomised controlled trial we will have asked the authors to provide the protocol and a CONSORT checklist. Other research designs should have the relevant checklist included (PRISMA, STARD, etc)
The correct checklist was used for qualitative research in lines 43-44

Additional points
In the abstract as well as in the general conclusion you state that insight from both the antenatal and postnatal perspectives can help to improve antenatal information provision. However, I do not seem to find HOW it might help. Also in the implications for clinicians section, you state HOW and What information should be provided is important to address without specification for implications. Perhaps you can guide clinicians a bith more on solutions for this How and What? Are birth plans routinely used for improving SDM at your hospital? Do you have continuity of care provider during antenatal care?

**Reviewer**	**2**
**Name**	**Simpson, Nigel**
**Affiliation**	**University of Leeds, Obstetrics and Gynaecology**
**Date**	**30-Mar-2025**
**COI**	**None**

You carried out a qualitative study exploring women's decision-making prior to and subsequent to birth. There was a small sample size as is not unusual in these studies, and this was sufficient for your needs - further participants would not have expanded the number of themes arising - but the inherent restriction in diversity and experience limited their scope of observation. I felt the paired pre-post format mitigated bias and affirmed the stability of the themes which had arisen. The study reinforces the desire of women to have consistent, disinterested, yet personalised information with which they may make informed decisions before and during labour.

VERSION 1 - AUTHOR RESPONSE

Reviewer	Reviewer’s comment	Author's response
Reviewer 1<br>Dr. Jeroen Van Dillen,<br>Radboud University Medical Center		
1.1	This study adds value by using repeat interviews to show how women’s views on decision-making change over time. It supports existing research on inconsistent information and social influences but adds depth by highlighting shifts in perception before and after birth. However, the unique contribution could be clearer. Emphasizing how repeat interviews provide new insights and distinguishing findings from past research would strengthen the discussion.	We thank the reviewer for this comment and the opportunity to expand on this study’s unique contribution. To address this comment, we have added the following paragraph to the discussion section of the manuscript:<br>In this study repeat interviews provided new insights into the interplay of information, communication and interactions surrounding shared decision-making as perceived by women before and after their experience of birth. Investigating women’s perceptions of decision-making during pregnancy and the postnatal period is more methodologically rigorous than exploration at a single time point. Post-partum quantitative and qualitative studies investigating women’s preferences, expectations and experiences of birth have been criticised as open to recall bias in successive systematic reviews (16,26,57). A 2021 systematic review of quantitative measures of the mismatch between expectations and experiences included only 11 prospective studies (16). As a method, the advantages of repeat interviews include that they can provide rich, contextualised and dynamic accounts of evolving and complex phenomena (58). This study adds new insights into the similarities and differences between women’s birth expectations and experiences of birth. The strength of similarities between antenatal and postnatal paired interviews in this study was unanticipated. Women’s priorities for information provided and views about influences on decision-making during pregnancy and after birth did not change. This was only evident because of the antenatal-postnatal paired interview design. The differences between antenatal and postnatal paired interview findings resonate with previous longitudinal studies that found experiential, embodied knowledge is gained by giving birth (59,60). The nuances in the postnatal findings could be addressed by the inclusion of women’s stories in decision-making information, alongside recognition and preparation for birth to not always go to plan. This study suggests women should be prepared for decision-making to be a dynamic process, whereby the impact of social influences, time constraints, and the role of healthcare professionals will evolve. In common with the

		conclusion of a systematic review of 13 qualitative and 19 quantitative studies of pain relief during labour this study shows women should make decisions based on what they would like to happen, whilst having an understanding of what might happen (26). One study of women's on-line chat forums suggests women commonly acknowledge the need to go with the flow (61).<br><br>References added to the manuscript<br>57. Gamble JA, Creedy DK. Women's request for a cesarean section: a critique of the literature. Birth. 2000 Dec;27(4):256-63. doi: 10.1046/j.1523-536x.2000.00256.x.<br>58. Murray S A, Kendall M, Carduff E, Worth A, Harris F M, Lloyd A et al. Use of serial qualitative interviews to understand patients' evolving experiences and needs BMJ 2009; 339 :b3702 doi:10.1136/bmj.b3702<br>61. Divall B, Spiby H, Nolan M, Slade P. Plans, preferences or going with the flow: An online exploration of women's views and experiences of birth plans. Midwifery. 2017 Nov;54:29-34. doi: 10.1016/j.midw.2017.07.020.
1.2	In the section implications for clinicians and policymakers:<br>This section is useful for clinicians and policymakers, emphasizing the need for personalized, well-timed information and the role of HCPs in decision-making. It highlights key barriers like time constraints but lacks clear solutions. Adding practical steps, like decision aids (e.g. birth plans?) or structured counselling, would improve its impact.	We thank the reviewer for this comment. We have added the following sentences and references to the end of the implications for clinicians and policymakers' section of the discussion:<br><br>There are several solutions. These include greater use of decision-aids, (68) birth plans (69) or structured counselling using core information sets. (56, 70-72)<br><br>References added to the manuscript<br>68. Black, M. Decision aid for planning mode of birth in routine antenatal care Plan A | Aberdeen Centre for Women's Health Research | The University of Aberdeen<br>69. Shareef N, Said P, Lamers S, Nieuwenhuijze M, de Vries M, van Dillen J. The contribution of birth plans to shared decision-making from the perspectives of women, their partners and their healthcare providers. PLoS One. 2024 Jun 26;19(6):e0305226. doi:10.1371/journal.pone.0305226.<br>70. Andrew Demetri AD, Danya Bakhbakhi, et al. What should be discussed when considering a vaginal birth? : A Delphi Study to develop a Core Information Set for vaginal birth. Authorea. February 07, 2025.<br>71. Eve Bunni VB, Carol Kingdon, et al. What should be discussed when considering an induction of labour? A Delphi study to develop a core information set for induction of labour. Authorea. March 02, 2025.<br>72. Carol Kingdon BG, Mahmoud Aljubeh, et al. What should be discussed when considering a caesarean birth?: A Delphi Study to develop a Core Information Set for caesarean birth. Authorea. March 02, 2025.
1.3	The evidence is presented scientifically and is clearly described, making it reliable and well supported.	We thank the reviewer for these comments. We read this as no response nor change required.
1.4	The aims of the study are clearly described and also answered, however the specific research question is not mentioned in the paper.	Following the journal style, we state our aim and objectives rather than our specific research question. Our research question was exploratory and purposively broad; How can women be best supported in shared decision-making for labour and birth? Figure 2, our overview of what women said they needed to know (antenatally),and wish they had known (postnatally) depicts our deductive findings. The orange section of Figure 2 and the three themes are the result of our inductive analysis of information accessed and influences on actual decisions made.
1.5	The study design is overall adequate and clearly described. However, the number of participants is somewhat low. A detailed description of the characteristics of these participants, as well as information on diversity and representativeness, would be a valuable addition.	Thank you for this comment. There are theoretical, empirical and personal differences of opinion about what constitutes an acceptable sample size in qualitative research.[1] Systematic review evidence shows data saturation in qualitative studies can be reached with between 9-17 participants in individual interviews.[2] We recruited 12 participants, purposively sampled for their diversity of socio-economic background, parity, previous birth experience and complexity of current pregnancy. Participant selection, sampling and sample size are explained in the method section. Participant characteristics are reported in Table 1 according to their parity and complexity of current pregnancy. We did not record socio-economic status or ethnic group as data. We have added this as a limitation in the manuscript.<br><br>Limitations of this study include recruitment from a single site, the inclusion of only one multiparous woman, and we did not record ethnicity or socio-economic data.<br><br>References included in the response, but not added to the manuscript

		1. Hennink M, Kaiser BN. Sample sizes for saturation in qualitative research: A systematic review of empirical tests. Soc Sci Med. 2022 Jan;292:114523.<br>2. Braun V, Clarke V. To saturate or not to saturate? questioning data saturation as a useful concept for thematic analysis and sample size Rationales. Qualitative Research in Sport, Exercise and Health 2021;13:201–16.
1.6	The inclusion and exclusion criteria for participants are clearly described, as well as the recruitment and selection process. However, a more detailed description of the participants is needed, especially considering that only 12 women were interviewed.<br><br>Also, in lines 49-54: Does low and high-risk pregnancy automatically mean different sociodemographic background? Can you explain further.	As described above, and acknowledged by Reviewer 2, 12 participants is an acceptable sample size for qualitative research when participants provide sufficient data, which Reviewer 1 acknowledges this study does. Unfortunately, we cannot report socio-economic status because we did not record it using an individual or area measure and to provide a more detailed narrative description of participants is problematic on the grounds of maintaining anonymity. We have added a sentence to the manuscript to address the second point. The reviewer is correct to question whether the categories low and high-risk pregnancy automatically mean different sociodemographic backgrounds. They do not. The manuscript has been changed to read:<br><br>We sought breadth and depth of views with representation from diverse women according to socio-economic background, parity, previous birth experience and complexity of current pregnancy. We approached both low and high-risk antenatal women in their first or subsequent pregnancy.
1.7	In lines 43-44, what do you mean by "interpretivist"?<br>Neutralizing personal biases about SDM is clearly described in the methods section under "Research Team and Reflexivity."<br>Section: Data Collection<br>How was the interview guide developed, and how was it structured? If relevant topics emerged during the interviews, how were these handled in relation to the interview guide and subsequent interviews?<br>Section: Data Analysis<br>How were the transcripts coded? Was a specific program used (e.g., Atlas.ti)?	Thank you for the opportunity to clarify what we mean by "interpretivist." The project is aligned to the interpretive tradition that originates from the work of Weber, Goffman and Garfinkel in Sociology. It seeks to understand meaning and significance from the perspective of the people who live within.<br><br>The interview guide was developed specifically for the purposes of the study informed by existing literature about antenatal education, preparing for birth and birth interventions, together with the recent proliferation of literature on shared decision making.<br><br>The interview guide structure reflects this trajectory in the literature, and the chronology of the study; with variants of the antenatal questions repeated postnatally. A copy of the interview guide is included in supplementary file 3. This was not a grounded theory study, so we did not extensively explore emergent topics.<br><br>Initial codes were generated by reading and re-reading transcripts, in chronological groups (timepoint 1, timepoint 2) and individually (antenatal, postnatal) by three authors who manually coded. Microsoft Excel was then used for data management, grouping and regrouping of codes. This is explained in the data analysis section of the manuscript and shown in supplementary file 4.
1.8	**Results - do they answer the research question? Are they credible and well-presented?** The results do answer the research aims of this study.<br><br>Theme 1: The theme effectively addresses the research aim by highlighting differences in antenatal and postnatal perceptions of information provision and decision-making. The use of direct quotes strengthens credibility, but clearer contrasts between pre- and postnatal insights would improve alignment with the research question.<br><br>Theme 2: The theme effectively addresses the research aim by illustrating how women navigate NHS and non-NHS information sources before and after birth. The findings are well-supported by quotes, highlighting perceived reliability issues and biases. However, a clearer comparison of how these perceptions shift antenatally vs. postnatally would strengthen alignment with the research question. Additionally, further exploration of	We thank the reviewer for their overall assessment that the results answer the aims of the study.<br><br>Theme 1: This has been addressed in the paragraph in response to point 1.1.<br><br>Theme 2: We did not probe how women reconcile conflicting information so are unable to perform additional analysis. However, we have added a sentence to the research recommendations as we agree with the reviewer a better understanding how women reconcile conflicting information would be interesting topic for further study.<br><br>Further research investigating how women reconcile conflicting information is also warranted given the myriad of information available.<br><br>Theme 3: We have added a sentence to the discussion (see 1.2) and conclusion (1.14).

	how women reconcile conflicting information could enhance the analysis.<br><br>Theme 3: The results effectively illustrate how timing and presentation of information influence decision-making before and after birth. The participant quotes add credibility and highlight key differences in information access, processing time, and perceived gaps. The findings provide a strong comparison between antenatal expectations and postnatal experiences. Overall, the section is well-structured, clear, and directly aligned with the research aim. Subtheme 3.3: It is unclear how to determine what information each woman wants and how to tailor it to individual needs. Adding insights on methods to personalize information, such as structured consultations, decision aids (such as birth plans), or patient feedback tools, would strengthen the discussion.	
1.9	Discussion: The discussion presents key findings well, but the comparison between antenatal and postnatal perceptions could be clearer. While some views stayed the same, the results show that birth experiences changed women's perspectives on social influences, time constraints, and the role of healthcare professionals in decision-making. The statement that there were "few differences" does not fully match the data, as some aspects of decision-making were reconsidered after birth. A clearer focus on how expectations (antenatal) compared to real experiences (postnatal) would better align with the study aim.	Our addition to the discussion in response to 1.1 addresses this comment (the second half in particular) which reads:<br><br>The differences between antenatal and postnatal paired interview findings resonate with previous longitudinal studies that found experiential, embodied knowledge is gained by giving birth (59,60). The nuances in the postnatal findings could be addressed by the inclusion of women's stories in decision-making information, alongside recognition and preparation for birth to not always go to plan. This study suggests women should be prepared for decision-making to be a dynamic process, whereby the impact of social influences, time constraints, and the role of healthcare professionals will evolve. In common with the conclusion of a systematic review of 13 qualitative and 19 quantitative studies of pain relief during labour this study shows women should make decisions based on what they would like to happen, whilst having an understanding of what might happen.(26) One study of women's on-line chat forums suggests women commonly acknowledge the need to go with the flow.(61)<br><br>Specifically in response to this comment we have amended the first sentence of the discussion to read:<br><br>In this qualitative antenatal-postnatal paired interview study on decision-making for labour and birth, we found similarities and differences in women's views about decision-making and information requirements when they were interviewed before and after delivery.
1.10	Strengths and weaknesses:<br>This section clearly presents the study's strengths and limitations. The paired interview design is a strong point, but the claim that no shift in views was found seems inconsistent with results showing some postnatal changes. The impact of COVID-19 on care needs is mentioned but not explored. Clarifying these points would improve accuracy.	We have addressed the first part of this comment in 1.1. and 1.9.<br><br>The reviewer is correct. The impact of COVID-19 on care needs is mentioned but not explored. We state in the manuscript that, as a limitation of the study, it is unclear to what extent their information and care needs were exacerbated by the COVID-19 pandemic. To improve accuracy, we have added to the manuscript in two places:<br><br>Results<br>All interviews took place between October 2021 and May 2022 during the NHS COVID-19 recovery period. COVID-19 was spontaneously mentioned in 7/22 interviews but there was no clear pattern to responses concerning decision-making. One woman felt there was a lack of antenatal education information leaflets; while another woman thought the shift to on-line antenatal education information was a good thing and very helpful. Two women talked about their experience of having COVID-19 during pregnancy. Another woman thought COVID-19 adjustments did not affect her care at all in comparison to her friend's birth experience during the first lockdown (March 2020). Two women felt their care was negatively affected during pregnancy. These women said they could not access a dating scan or external cephalic conversion. One further woman mentioned she wanted to avoid induction of labour because of restrictions on birth partner visiting. It is unclear if this was the Trust policy at the time of her birth or based on other women's accounts from the first few months of the pandemic response.

		Discussion<br>Existing research shows pandemic-driven changes impacted NHS maternity and neonatal service delivery, user experience, and staff wellbeing in different ways, at different times.(62) While not the focus of this study, COVID-19 was spontaneously mentioned in 7/22 interviews, but not consistently about decision-making.<br><br>Reference to be added to the manuscript<br>62. Neal S, Stone L, Moncrieff G, Matthews Z, Kingdon C, Topalidou A, Balaam MC, Cordey S, Crossland N, Feeley C, Powney D, Sarian A, Fenton A, Heazell AEP, de Jonge A, Severns A, Thomson G, Downe S. Assessing safe and personalised maternity and neonatal care through a pandemic: a case study of outcomes and experiences in two trusts in England using the ASPIRE COVID-19 framework. BMC Health Serv Res. 2023 Jun 22;23(1):675. doi: 10.1186/s12913-023-09669-0.
1.11	The references are up-to-date and relevant, with no apparent omissions.	We read this as no response nor change required.
1.12	Abstract: In the design section, consider specifying the exact timing of the first and second interviews.Data Collection: Include details on how participants were recruited.<br>Data Analysis: Provide more information on how the thematic analysis was conducted. Did you use any specific tools or software?	We have amended the Abstract as requested to include further detail about timing of interviews, recruitment and analysis, while trying not to add too many words. It now reads:<br><br>Design: Qualitative interview study using repeat interviews at two-time points; during pregnancy (≥13 weeks gestation); and after birth (≥6 weeks).(antenatal and postnatal).<br>Participants: Pregnant women accessing antenatal care were purposively sampled and recruited antenatally by community midwives to ensure representation from different socio-demographic groups, with diverse experiences of low and high-risk care.<br>Data analysis: Interviews were audio recorded, transcribed verbatim, and qualitative thematic analysis was conducted using Braun and Clarke's six-stage process.<br><br>Further detail about how we conducted the thematic analysis is available in Supplementary file 4. We used Microsoft Excel for data management and analysis as stated in the methods section of the manuscript.
1.13	The correct checklist was used for qualitative research in lines 43-44	We read this as no response nor change required.
1.14	In the abstract as well as in the general conclusion you state that insight from both the antenatal and postnatal perspectives can help to improve antenatal information provision. However, I do not seem to find HOW it might help.<br><br>Also in the implications for clinicians section, you state HOW and What information should be provided is important to address without specification for implications.<br>Perhaps you can guide clinicians a bit more on solutions for this How and What? Are birth plans routinely used for improving SDM at your hospital? Do you have continuity of care provider during antenatal care?	Thank you for this comment. In addition to the sentences added to the discussion section as described above (1.2), we have added the following sentence to the conclusion sections of the abstract and the manuscript.<br><br>Antenatal interventions that warrant further research include birth plans, decision aids, and structured counselling using core information sets.<br><br>We have also amended the last sentence of the abstract to read: Insights from both antenatal and postnatal perspectives will help inform their development.<br><br>It was beyond the scope of this study to collect specific data on the use of birth plans and continuity of carer to improve SDM. This is an interesting area for further research. The continuity of carer model is part of routine NHS antenatal care.
Reviewer 2<br>Dr. Nigel Simpson, University of Leeds		
	You carried out a qualitative study exploring women's decision-making prior to and subsequent to birth. There was a small sample size as is not unusual in these studies, and this was sufficient for your needs - further participants would not have expanded the number of themes arising - but the inherent restriction in diversity and experience limited their scope of observation. I felt the paired pre-post format mitigated bias and affirmed the	We thank the reviewer for these comments. We read this as no response nor change required.

	stability of the themes which had arisen. The study reinforces the desire of women to have consistent, disinterested, yet personalised information with which they may make informed decisions before and during labour.	

For clarity, where appropriate, we have also reduced our use of the SDM acronym throughout the manuscript.
All changes are tracked in the uploaded manuscript.
